# Transient dynamic phenotypes as criteria for model discrimination: fold-change detection in *Rhodobacter sphaeroides* chemotaxis

**DOI:** 10.1098/rsif.2012.0935

**Published:** 2013-03-06

**Authors:** Abdullah Hamadeh, Brian Ingalls, Eduardo Sontag

**Affiliations:** 1 Department of Electrical and Computer Engineering, 94 Brett Road, Rutgers, The State University of New Jersey, Piscataway, NJ 08854-8058, USA; 2 Department of Mathematics, Hill Center, 110 Frelinghuysen Road, Rutgers, The State University of New Jersey, Piscataway, NJ 08854-8019, USA; 3 Department of Applied Mathematics, University of Waterloo, 200 University Avenue West, Waterloo, Ontario, Canada N2L 3G1

**Keywords:** chemotaxis, model discrimination, *Rhodobacter sphaeroides*, fold-change detection

## Abstract

The chemotaxis pathway of the bacterium *Rhodobacter sphaeroides* shares many similarities with that of *Escherichia coli*. It exhibits robust adaptation and has several homologues of the latter's chemotaxis proteins. Recent theoretical results have correctly predicted that the *E. coli* output behaviour is unchanged under scaling of its ligand input signal; this property is known as fold-change detection (FCD). In the light of recent experimental results suggesting that *R. sphaeroides* may also show FCD, we present theoretical assumptions on the *R. sphaeroides* chemosensory dynamics that can be shown to yield FCD behaviour. Furthermore, it is shown that these assumptions make FCD a property of this system that is robust to structural and parametric variations in the chemotaxis pathway, in agreement with experimental results. We construct and examine models of the full chemotaxis pathway that satisfy these assumptions and reproduce experimental time-series data from earlier studies. We then propose experiments in which models satisfying our theoretical assumptions predict robust FCD behaviour where earlier models do not. In this way, we illustrate how *transient dynamic phenotypes* such as FCD can be used for the purposes of discriminating between models that reproduce the same experimental time-series data.

## 1. Introduction

Dynamic models of biological mechanisms are meaningful if they can explain experimental data, can make *a priori* predictions of biological behaviour, and are liable to invalidation through testing.

Although several competing models of a given mechanism can often be made to reproduce experimental data through parameter tuning, in many cases, it is possible to discriminate between such models by comparing the experimentally observed output response and the simulated response to judiciously designed perturbations. This paper is a study of the use of a particular *transient dynamic phenotype* for the purposes of model discrimination. We define transient dynamic phenotypes to be distinctive, qualitative, dynamic and transient output responses that are robustly maintained under a range of experimental conditions.

An example of a transient dynamic phenotype is adaptation, where a system's output at steady states corresponding to constant input stimuli is independent of the magnitude of the input. It has been shown that integral control is a structural feature that can be responsible for this behaviour [1]. Weber's law, whereby a system exhibits the same maximal amplitude in its response to two different inputs that are positive linear scalings of each other [2], is another example of a transient dynamic phenotype.

This study deals with a third transient dynamic phenotype, termed fold-change detection (FCD) [2] or scale invariance. A system is said to exhibit FCD if its output responses to two different input stimuli that are positive linear scalings of each other are identical, making this a property that includes, but is stronger than, Weber's law.

In references [2,3], it was predicted that the chemotaxis system of *Escherichia coli*, modelled in [4], would exhibit the FCD property, and these predictions were later confirmed as accurate [5]. The key assumption of this model, which leads to FCD, is the allosteric signalling structure of the methyl-accepting chemotaxis protein receptors.

Although significantly more complex, the chemotaxis system of the bacterium *Rhodobacter sphaeroides* has many similarities to that of *E. coli*. It features two, rather than one, sensory clusters; one at the cell membrane and the other in the cytoplasm. While the membrane cluster, as in *E. coli*, detects external ligand, it is as yet unknown exactly what the cytoplasmic cluster senses [6]. Besides detecting internalized ligand concentrations, it may also sense internal signals, such as signals reporting the cell's metabolic state. This bacterium also has multiple homologues of the *E. coli* chemotaxis proteins, which play roles similar to those found in the latter, although the exact structure of their connectivity with the two sensory clusters and the flagellum is not known with certainty. The CheA homologues transduce the receptor activity to the other chemotaxis proteins through phosphotransfer, the CheR and CheB homologues, respectively, methylate and demethylate receptors, whereas the CheY proteins are believed to have a role in varying the stopping frequency of the bacterium's single flagellum [7].

Recent studies have used a model invalidation technique to suggest possible connectivities for the CheY proteins [8] and the CheB proteins [9]. However, upon simulation, it becomes evident that these models do not exhibit the FCD behaviour observed in *E. coli*. Recent experimental results [10] suggest that *R. sphaeroides* does in fact show constant adaptation times in response to scaled step changes in its sensed ligand concentration, which is in line with, though not definitive proof of, FCD behaviour in this bacterium; the latter would require evidence that shows the entire shape of the bacterium's chemotactic response curve is unchanged when it is subject to such step inputs. Furthermore, experimental results show that this evidence for FCD is observed in bacteria grown under a variety of different conditions, each of which is likely to lead to different cell architectures, protein expression levels and stoichiometries.

In the light of this preliminary evidence for FCD, here we model the dynamics of the two *R. sphaeroides* receptor clusters using the MWC allosteric model [11] that has been used to model the receptor activity in *E. coli* in earlier studies [4,12,13]. We present a theorem that shows that if this is an accurate model of the receptor dynamics, then the receptor activities will exhibit FCD. Moreover, this observed behaviour is robust to the connectivity between the chemotaxis proteins, the receptors and the flagellum. It is also robust to parametric variations, in line with the observed evidence for FCD in bacteria grown in different conditions. To illustrate these points, we construct two models of the integrated *R. sphaeroides* chemotaxis pathway based on our receptor dynamics assumptions, with each model featuring a different internal connectivity. We show that these two models are capable of reproducing previously published experimental data. In addition, we show, both analytically and through simulations, that these models, along with a slightly modified version of the model presented in [10], display exact FCD in their flagellar responses in certain ligand concentration ranges. Next, we suggest a series of experiments that can be used to test whether the models we present here are accurate compared with previously published models, based on whether or not the flagellar response exhibits FCD.

Because the methylation dynamics of these models can reproduce the transient dynamic phenotype of FCD, we argue that they are more accurate representations of the actual biochemistry than previously published models. This work therefore makes the case that qualitative dynamic behaviour could be a powerful property to test when discriminating between competing models. A systematic method for model discrimination using this approach would start with the construction of a dynamic model that explains experimental data. The next step would be to use the model to mathematically identify experimentally implementable conditions under which the system can be expected to exhibit a certain transient dynamic phenotype. The final step would be to experimentally implement those conditions and to compare the measured results against what is predicted *in silico*. In this way, transient dynamic phenotypes can be used to discriminate between models that explain experimental data equally well.

### 1.1. Background

**Figure 1.**
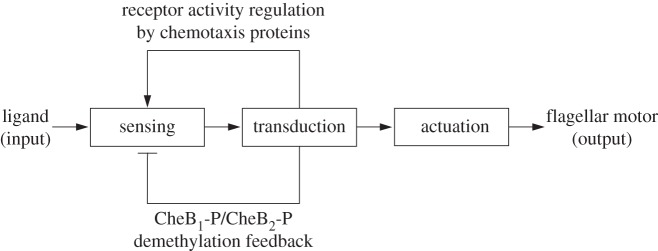
Schematic of the *R. sphaeroides* chemotaxis pathway.

**Figure 2.**
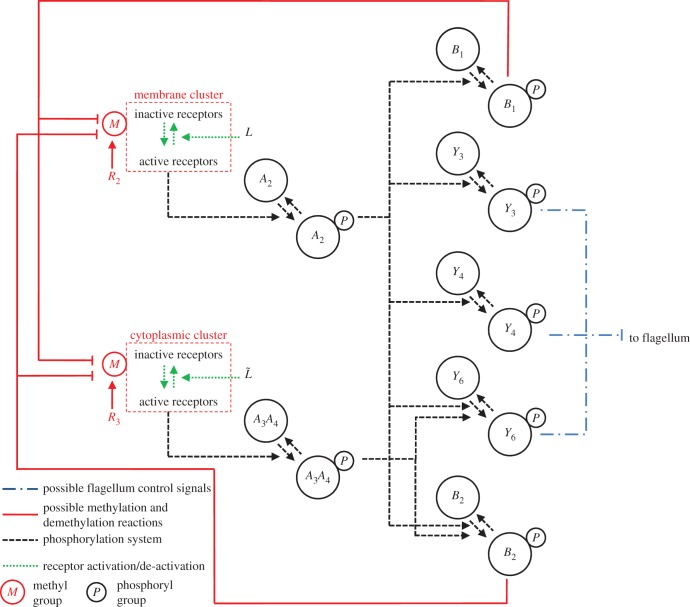
The *R. sphaeroides* signalling network. (Online version in colour.)

We can decompose the *R. sphaeroides* chemotaxis pathway into three modules, as illustrated in figure 1. The sensing module includes two receptor clusters. One of these resides at the cell membrane and senses the concentration of external ligands *L*, as illustrated in figure 2. The other cluster resides within the cytoplasm and measures an internalized ligand concentration *L̃*. Henceforth, the notation symbol ‘tilde’ will be used to denote signals associated with the cytoplasmic cluster.

The dynamics of the two receptor clusters are modelled as two first-order systems. The membrane receptor cluster is assumed to have state *m* (its average receptor methylation level) and output *a* (the receptor activity level). Similarly, the cytoplasmic cluster has average methylation level *m̃* as its state and its activity level *ã* as its output. The state-space representation of this system is then1.1
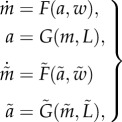
where *w*, *w̃* are functions of the concentrations of the phosphorylated chemotaxis proteins within the cell. These functions represent the interactions between the internal state of the cell and the receptors. For example, *w* and *w̃* can represent the demethylation of the receptors by the proteins CheB_1_, CheB_2_ or their methylation by the proteins CheR_2_,CheR_3_.

The cytoplasmic cluster is believed to integrate the extracellular ligand concentration *L* with internal cell signals, although the precise mechanism through which this is achieved and the exact set of signals it detects is unknown [6]. To allow for a wide range of possible interactions between the cytoplasmic cluster on the one hand and, on the other hand, the externally sensed ligands *L* and the phosphorylated chemotaxis proteins, collectively represented by a signal *u*, we let signal *L̃* (the signal sensed by the cytoplasmic cluster) be the output of a dynamical system given in assumption A.1 in the appendix. With this assumption, the internalized ligand concentration *L̃* can represent a variety of signals, including, for example, a static map that combines the externally sensed ligands *L* with the internal chemotaxis protein signals *u*, or it can be a phase-delayed version of *L* or even, to allow for a degree of possible cooperativity, a power of *L*.

In the transduction subsystem of figure 1, the activity of the two sensing clusters is conveyed to the chemotaxis protein system through an acceleration of the auto-phosphorylation rates of the CheA proteins. The auto-phosphorylation of the chemotaxis protein CheA_2_ is accelerated by the membrane cluster activity, whereas that of the CheA_3_A_4_ complex is catalysed by cytoplasmic cluster activity (as shown in figure 2). The proteins CheY_3_, CheY_4_, CheY_6_, CheB_1_ and CheB_2_ all compete for phosphoryl groups from CheA_2_, whereas CheB_2_ and CheY_6_ do so from CheA_3_A_4_. The reaction rates for all of these phosphorylations are given in references [8,14]. We represent this phosphotransfer network as a general nonlinear system, with state vector
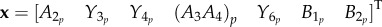
the individual states being the concentrations of the phosphorylated chemotaxis proteins. The transduction system takes as its inputs the receptor activities *a*, *ã*:1.2
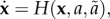
where *H*(**x**, *a*, *ã*) is given in the electronic supplementary material.

**Figure 3.**
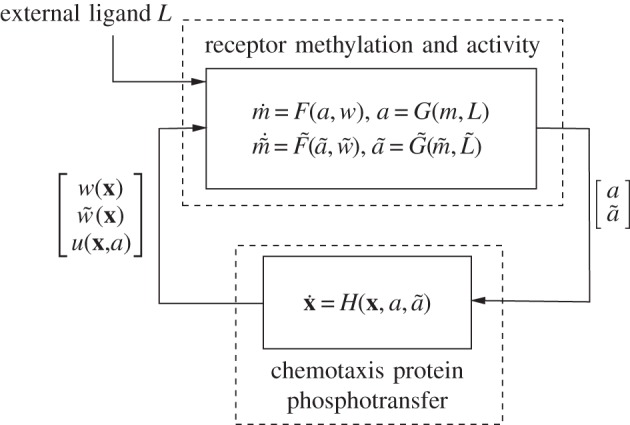
The interconnection of the receptors' sensing dynamics with the phosphotransfer network.

The outputs of this system are signals *w*(**x**), *w̃*(**x**), *u*(**x**,*a*), which feed back into the sensing subsystem, as described earlier. The interconnection of the phosphotransfer network (1.2) with the receptor dynamics is illustrated in figure 3, and the interconnection between the two subsystems can thus be written as1.3
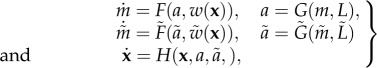
where the dynamics governing *L̃* are given in assumption A.1 (see appendix).

As shown in figure 2, the protein CheY_6_-P, possibly acting together with one or both of CheY_3_-P and CheY_4_-P, is believed to bind with the flagellar motor proteins to inhibit the flagellar rotation rate [7] (thus effectively coupling the signal transduction system to the actuation system), though the precise mechanism through which this is achieved is unknown. An additional uncertainty lies in the demethylation connectivity between the CheB proteins and the two receptor clusters, though these questions have been the subjects of several studies [8,9,15]. Although how the CheB and CheY proteins interact with the sensing and actuation modules is not known for certain, it will be shown later in the study that, under some mild assumptions, FCD can be exhibited by the bacterium, regardless of the exact structure of these connectivities.

### 1.2. A Monod–Wyman–Changeux model of receptor dynamics

We use a Monod–Wyman–Changeux (MWC)-type allosteric model for the receptor activities [11]. Such models have been proposed for several bacterial chemotactic systems and have been found to be consistent with experimental data [4,12,13,16]. The main assumptions of the model are that receptors are either active or inactive, and that ligands have a higher affinity for inactive receptors than for active receptors. Respectively, we denote by *a*(*t*) and *ã*(*t*) the probabilities at time *t* of a transmembrane and cytoplasmic receptor being active. For each receptor, this probability can be approximated by the ratio of the Boltzmann factor of the active state to the sum of the Boltzmann factors of all the states. Therefore, if, at time *t*, the free-energy state of the membrane receptors is *E*_A_ when active and *E*_I_ when inactive, then the activity of the membrane receptors is approximated by1.4
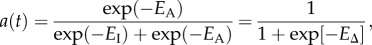
where *E*_*Δ*_ = *E*_I_−*E*_A_ is the free energy difference between the active and inactive states.

Similarly, for the cytoplasmic receptors, the activity *ã*(*t*) is dependent on their free-energy states when active and inactive, respectively *Ẽ*_A_ and *Ẽ*_I_:1.5
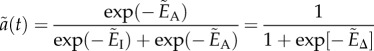
with *Ẽ*_*Δ*_ = *Ẽ*_I_−*Ẽ*_A_. The functions *E*_*Δ*_ and *Ẽ*_*Δ*_ are assumed to have the same structure and take the form *E*_*Δ*_ =−*N*[*g*_*m*_(*m*) + *g*_L_(*L*)] and *Ẽ*_*Δ*_ =−*Ñ*[*g̃*_*m*_(*m̃*) + *g̃*_L_*L̃*)]. The functions *g*_*m*_, *g̃*_*m*_ are dependent on the methylation state of their respective receptors, whereas the functions *g*_L_, *g̃*_L_ quantify the effect of ligand binding on the receptor-free energy difference of the receptors. The parameters *N*, *Ñ* represent the degree of dimerization in the receptor cluster, and are taken to be constants in references [4,5] and in our models. Following earlier studies [4,5,16], we make the assumption that each of *g*_*m*_, *g̃*_*m*_ is affinely dependent on the methylation state of its respective receptor cluster:
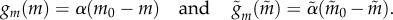
The constants *α*, 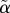 quantify the sensitivity of the free energy differences *E*_*Δ*_, *Ẽ*_*Δ*_ to changes in the average receptor methylation level, whereas *m*_0_, *m̃*_0_ are parameters that represent biases in *E*_*Δ*_, *Ẽ*_*Δ*_ such that *αm*_0_ and 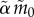 are the energy differences between the active and inactive membrane and cytoplasmic receptors, respectively, when the average receptor methylation level and the sensed ligand concentration are both zero.

The binding of ligands to receptors leads to a loss of ligand translational entropy, proportional to the logarithm of the free ligand concentration [4,13]. Owing to the greater affinity of ligands to inactive receptors, this loss is greater in the case of ligands binding to active receptors. We denote the dissociation constants between ligands and active transmembrane (cytoplasmic) receptors by *K*_A_ (*K̃*_A_), and between ligands and inactive transmembrane (cytoplasmic) receptors by *K*_I_ (*K̃*_I_), with *K*_A_ ≫ *K*_I_ and *K̃*_A_ ≫ *K̃*_I_ owing to the different affinities. To the authors' best knowledge, the values of these constants have not been measured for *R. sphaeroides*, and therefore, from the *E. coli* chemotaxis literature, we adopt the values *K*_I_ = *K̃*_I_ = 18 µM, *K*_A_ = *K̃*_A_ = 3 µM from [4]. As in [13], the change in receptor free energies owing to ligand binding to active transmembrane and cytoplasmic receptors is then, respectively, −ln(*L*/*K*_A_) and−ln(*L̃*/*K̃*_A_). On the other hand, the change in receptor free energy owing to ligand binding to inactive transmembrane and cytoplasmic receptors is, respectively, −ln(*L*/*K*_I_) and −ln(*L̃*/*K̃*_I_). The effect of this on the free energy differences *E*_*Δ*_, *Ẽ*_*Δ*_ between active and inactive receptors can be characterized, as in recent studies [4,13], as
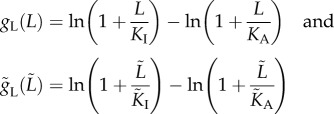
for each cluster, respectively. Owing to the differences in affinities, we note that *g*_L_(*L*) and *g*_L_(*L̃*) are increasing functions of *L* and *L̃*, respectively, which means that *a*(*t*) and *ã*(*t*) are decreasing functions of *L* and *L̃*, respectively. The greater affinity of ligands for inactive receptors therefore has the effect of shifting the receptors towards the inactive state.

Note that in the ligand concentration range *K*_I_ ≪ *L* ≪ *K*_A_ and *K̃*_I_ ≪ *L̃* ≪ *K̃*_A_, the receptor activities can be approximated by1.6
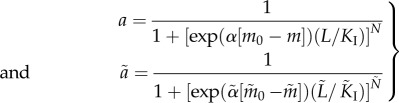


## 2. Main results

Following similar definitions in the literature [2,3], we say that the *R. sphaeroides* chemotaxis system (1.3) exhibits adaptation if, at steady states corresponding to any constant ligand input signal *L*, its receptor activities *a*, *ã* are independent of *L*. Furthermore, system (1.3) exhibits FCD in response to a sensed ligand input signal *L*(*t*) if its receptor activity output responses *a*(*t*), *ã*(*t*), initially at steady states corresponding to constant inputs *L*(0), are independent of linear scalings *p* > 0 of the input *L*(*t*). Theorem A.2 in the appendix presents the main theoretical result of this study, that the assumptions placed on the bacterium's chemosensory dynamics in §§1.1 and 1.2 lead to both adaptation and FCD in the ligand range *K*_I_ ≪ *L* ≪ *K*_A_ and *K̃*_I_ ≪ *L̃* ≪ *K̃*_A_. In particular, to prove that system, (1.3) will display the adaptation that is necessary for FCD, theorem A.2 uses approximation (1.6) and the fact that the feedback structure of (1.3) is, as in classical integral control, such that functions *F*(*a*,*w*(**x**)), *F̃*(*ã*,*w*(**x**)) do not explicitly depend on *m*, *m̃*. In general however, FCD does not necessarily require integral feedback and, as is discussed in [2,3], it can be observed in systems that adapt through other mechanisms, such as the incoherent feed-forward loop.

Note that the chemotaxis protein phosphorylation network (1.2) takes as its sole inputs the signals *a* and *ã*. For this reason, the above definition of FCD implies that if the system (1.3) exhibits FCD in its activities, it also exhibits FCD in the concentration of its phosphorylated chemotaxis proteins (the elements of the vector **x**). The bacterium's flagellar behaviour would also be expected to exhibit FCD as the flagellum rotation rate is a function of the phosphorylated CheY_3_, CheY_4_ and CheY_6_ concentrations.

### 2.1. Three *R. sphaeroides* chemotaxis models

There are several integrated *R. sphaeroides* chemotaxis pathway models in the literature [8,9,15]. In this section, we present two new models (models I and II), differing from the previous ones in that their receptor dynamics are of the form (1.3) and satisfy the MWC model given in §1.2. The complete set of ODEs and parameters that describe the two models are given in the electronic supplementary material. The parameters of both models were obtained by fitting to experimental data available in [9]. The two models were subsequently able to reproduce the gene deletion data in references [8,9]. We additionally review a third model (model III) from [10], and we show that slightly modifying it so that it satisfies (1.3), and §1.1 is sufficient to make the model show exact FCD.

Each of the models presented satisfies the assumptions of §1.2 and thereby exhibits FCD in the ligand range *K*_I_ ≪ *L* ≪ *K*_A_ and *K̃*_I_ ≪ *L̃* ≪ *K̃*_A_. As discussed in §1.1, the CheB_1_, CheB_2_ demethylation feedback is represented by the functions *w*(**x**) and *w̃*(**x**) in (1.3). In the proof of theorem A.2, we show that models of the form (1.3) exhibit FCD for any feedback structure *w*(**x**) and *w̃*(**x**) (excluding the trivial case where there is no feedback because such a system would not show an adaptive response in the first place). However, for the purposes of modelling, the demethylating feedback structure for models I and II is restricted to that in [9], which proposed an asymmetric connectivity wherein CheB_2_ demethylates both clusters and CheB_1_ demethylates the transmembrane cluster. The fact that the exact feedback structure does not impact the model's ability to detect fold changes will be illustrated by the fact that model III, from [10], has a different feedback structure and yet is still able to show FCD.

The structural differences between the models I and II lie in the signal *L̃*, which captures how external ligands are transduced to the cytoplasmic cluster. This also illustrates the point that FCD is conserved under changes in internal connectivities with the assumptions we make in §1.2.

Following the earlier studies [9,17], the models we present are such that the CheB proteins demethylate only active receptors, whereas CheR proteins methylate only inactive receptors and operate at saturation. The CheR_2_ and CheR_3_ protein concentrations are therefore constant and normalized to 1 µM each, as in [9]. Denoting by *R*_2_, *R*_3_ the concentrations of CheR_2_ and CheR_3_ and by *B*_1_*p*__, *B*_2_*p*__ the concentrations of phosphorylated chemotaxis proteins CheB_1_, CheB_2_, mass action kinetics give the following general form for *F*, *F̃* in (1.3)2.1
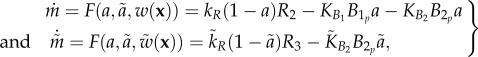
where *k*_*R*_, *k̃*_*R*_ > 0 are methylation and *k*_*B*_1__, *k*_*B*_2__, *k̃*_*B*_2__ > 0 demethylation rate constants. The probabilities of activity *a*, *ã* given by (1.4) and (1.5).

The experimentally measured output which was used to fit the model is the rotation frequency *f* of the tethered flagellum. As shown in figure 2, the CheY proteins control the rotation of the flagellum, and this is believed to happen through inhibitory binding [8]. The measured rotation frequencies to which we fit our models varied between 0 Hz and a maximum of approximately 8 Hz. As discussed in [9], this maximum was very rarely exceeded, and is therefore assumed to be a physical limit on how fast the tethered flagellum can rotate (in nature, the untethered flagellum rotates at frequencies much higher than 8 Hz). For this reason, the rotation frequency is modelled as the Hill function
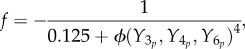
where2.2
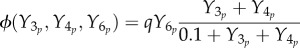
(the negative sign denotes anti-clockwise rotation). In this way, *f* varies between 0 and 8 Hz, and decreases with increased concentrations of phosphorylated CheY proteins. The Hill coefficient of 4 follows from the earlier work in [9]. To the authors' best knowledge, the degree of cooperativity between the CheY proteins that bind to the motor proteins in *R. sphaeroides* has not yet been experimentally deduced, although this quantity has been shown to be as high as 10 for *E. coli* [18].

We used the simulated annealing optimization algorithm in the Matlab systems biology toolbox to fit the parameters *k*_*R*_, *k̃*_*R*_, *k*_*B*_1__, *k*_*B*_2__, *k̃*_*B*_2__ in (2.1) and *q* in (2.2) to tethered cell assay data from [9]. The values of *α*, 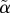 (from §1.2) are fixed to 2 kT and not fitted to the data because a scaling of these parameters by a factor *ε* is equivalent to respectively scaling by 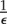 the fitted parameter sets *k*_*R*_, *k*_*B*_1__, *k*_*B*_2__ and *k̃*_R_, *k̃*_*B*_2__ (this can be seen by considering a change in variables that scales the methylation levels *m*, *m̃* in (1.4) and (1.6) by *ε*). The parameters *m*_0_, *m̃*_0_ were also fixed to 5 because they appear only in (1.6) in the constants e^*αm*_0_^, 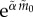 which scale the inputs *L*, *L̃* and have no bearing on the adaptation response owing to FCD.

#### 2.1.1. Model I

**Figure 4.**
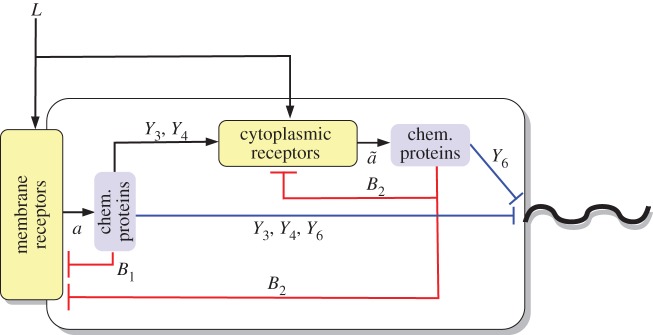
Schematic of model I. (Online version in colour.)

*Model structure*. In this model, the cytoplasmic receptors are assumed to sense internalized ligands, the concentrations of which are dependent on the external ligand concentration *L*. At the same time, as in [9], we assume there to be some interaction between the chemotaxis proteins CheY_3_, CheY_4_ and the cytoplasmic cluster, and the function *g̃*_L_(*L̃*) takes as its input *L̃* = 10*L*/(10 + *Y*_3_*p*__ + *Y*_4_*p*__). Schematic of this model is shown in figure 4.

**Figure 5.**
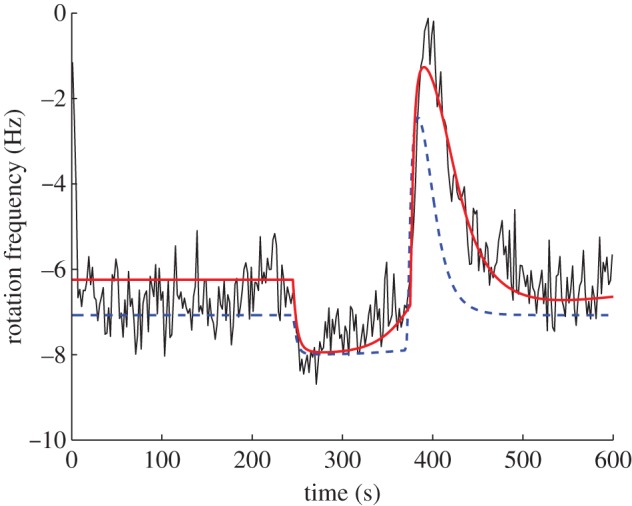
Simulation of model I (red) in response to a step rise (at 245 s) and fall (at 375 s) in the ligand level *L* from *L* = 0 to *L* = 100 and back to *L* = 0, with a tethered cell assay (black). The dashed blue trace is a simulation of the previously published model in [9] subject to the same ligand input. (Online version in colour.)

A simulation of the model together with the tethered cell trace to which the model was fitted is shown in figure 5. For comparison, figure 5 additionally shows a simulation (with the same ligand input) of the model suggested in [9], which was fitted to the same tethered cell assay. The root mean-squared error between the output of model I and the tethered cell assay is 0.77, which compares favourably to the corresponding error for the model in [9], which is 1.18.

**Figure 6.**
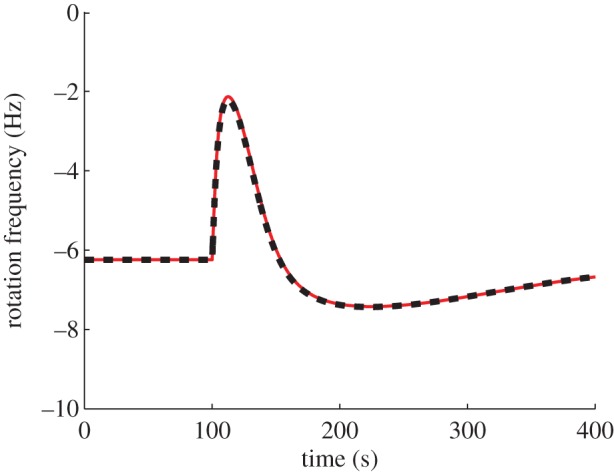
Model I output in response to step changes in *L* from *L* = 1000 to 200 µM (red) and from *L* = 500 to 100 µM (black). (Online version in colour.)

This model would be expected to exhibit FCD in the ligand ranges *K*_I_ ≪ *L* ≪ *K*_A_ and *K̃*_I_ ≪ *L̃* ≪ *K̃*_A_. The latter range is equivalent to
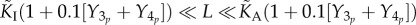
and because the total amounts of intracellular CheY_3_ and CheY_4_ (phosphorylated and un-phosphorylated) are 3.2 and 13.2 µM, respectively, then according to this model, simulations should show FCD in the range 2.64 *K̃*_I_ ≪ *L̃* ≪ *K̃*_A_. Figure 6 shows that this is indeed the case, with identical output traces obtained for the step changes in *L* from *L* = 1000 to 200 µM and from *L* = 500 to 100 µM.

*Model parameters*:
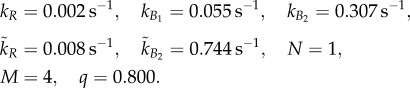


#### 2.1.2. Model II

**Figure 7.**
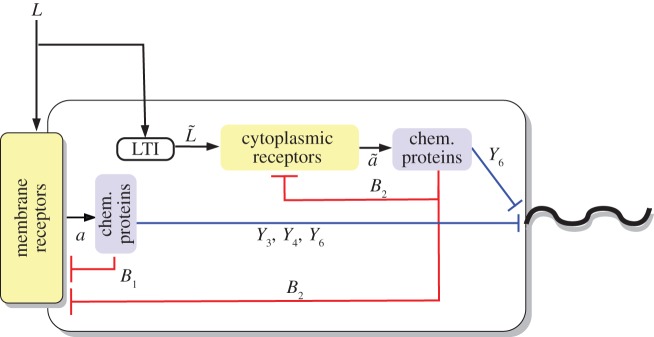
Schematic of model II. (Online version in colour.)

**Figure 8.**
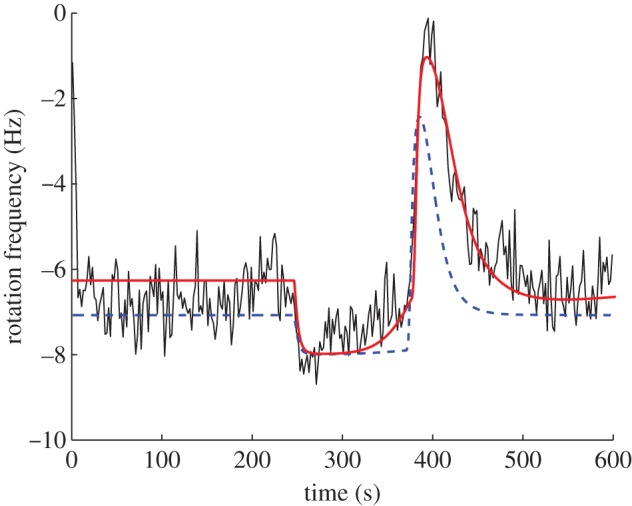
Simulation of model II (red) in response to a step rise (at 245 s) and fall (at 375 s) in the ligand level *L* from *L* = 0 to *L* = 100 and back to *L* = 0, with a tethered cell assay (black). The dashed blue trace is a simulation of the previously published model in [9] subject to the same ligand input. (Online version in colour.)

*Model structure*. Here, the model's internally sensed ligands *L̃* are related to *L* via the linear time invariant filter
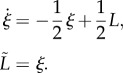
While the ligand concentrations *L*, *L̃* modify receptor activities, the cytoplasmic receptors are otherwise unregulated by internal cell signals, and therefore *L̃* is not a function of *u*. Schematic is illustrated in figure 7, and a simulation of the model together with the tethered cell trace to which the model was fitted is shown in figure 8. For comparison, figure 8 additionally shows a simulation (with the same ligand input) of the model suggested in [9], which was fitted to the same tethered cell assay. The root mean-squared error between the output of model II and the tethered cell assay is 0.75, which, as with model I, also compares favourably with the corresponding error for the model in [9], which is 1.18.

**Figure 9.**
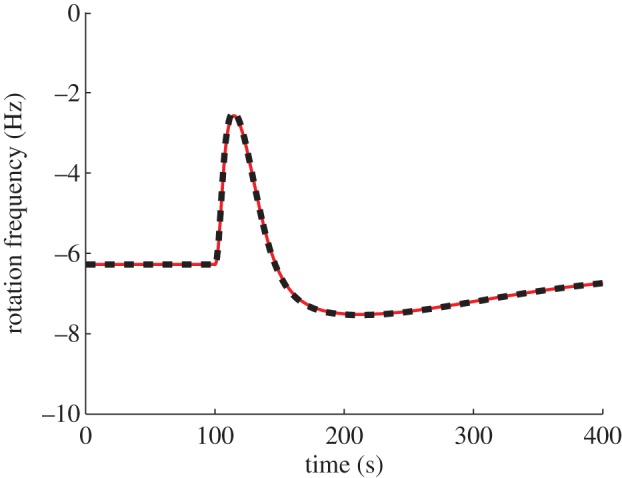
Model II output in response to step changes in *L* from *L* = 1000 to 200 µM (red) and from *L* = 500 to 100 µM (red). (Online version in colour.)

Note that if *L* were to undergo a step change from *L* = *L*_*a*_ µM to *L* = *L*_*b*_ µM, and if the system is initially at steady state (where *L̃*(0) = *L*_*a*_), then *L̃* would remain confined to the set [*L*_*a*_, *L*_*b*_). Therefore, for such a step change, *K*_I_ ≪ *L* ≪ *K*_A_ implies that *K̃*_I_ ≪ *L̃* ≪ *K̃*_A_. Figure 9 shows that simulations of this model do show FCD in this input range, with similar output traces obtained for the step changes in *L* from *L* = 1000 to 200 µM and from *L* = 500 to 100 µM.

*Model parameters*:
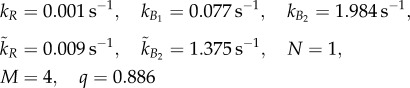


#### 2.1.3. Model III

**Figure 10.**
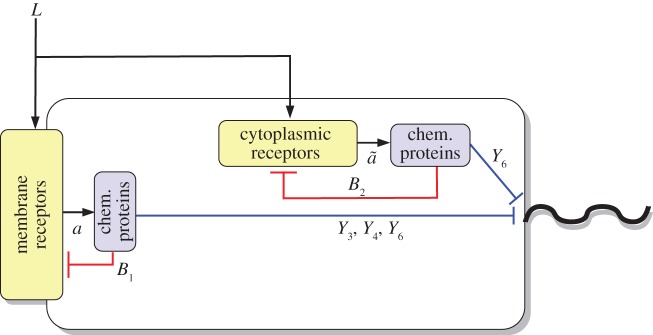
Schematic of model III. (Online version in colour.)

**Figure 11.**
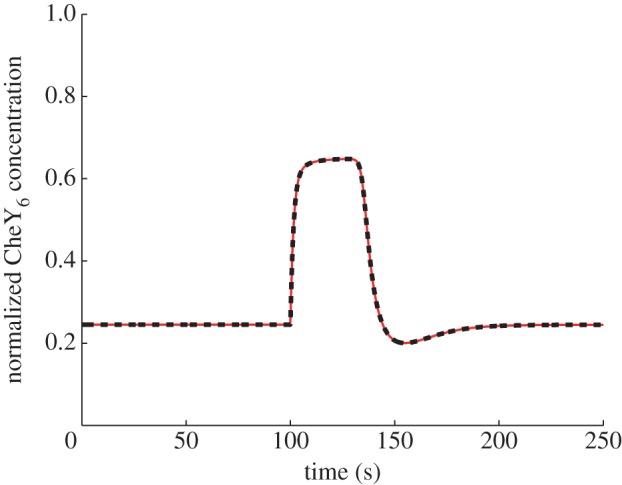
Simulation of model III with 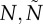 constant, showing normalized CheY_6_ concentration in response to step changes in *L* from *L* = 1000 to 200 µM (red) and from *L* = 500 to 100 µM (black). (Online version in colour.)

*Model structure*. [10], which presents evidence for FCD in *R. sphaeroides*, also presents a chemotaxis dynamical model, schematic of which is shown in figure 10. Compared with models I and II, this system has a different CheB_1_/CheB_2_ feedback structure (CheB_1_ demethylates membrane cluster receptors, CheB_2_ demethylates cytoplasmic receptors), it includes no interaction between the CheY_3_, CheY_4_ proteins and the receptors as in model I, and the ligand signals are conveyed to the cytoplasmic cluster quickly, via a static map, unlike model II. In addition, the degrees of receptor cooperativity, *N*, *Ñ* are modelled as functions of ligand concentration. With this latter feature, simulations of this model in [10] show approximate FCD. If the quantities *N*, *Ñ* are made constant however, the model in [10] is of the form (1.3) and satisfies the assumptions of §1.2. As expected from theorem A.2, with this modification (*N* = *Ñ* = *a*_0_, where *a*_0_ = 17.5 from [10]), the model displays exact FCD under approximation (1.6), as shown in figure 11.

### 2.2. Future experiments for model invalidation

**Figure 12.**
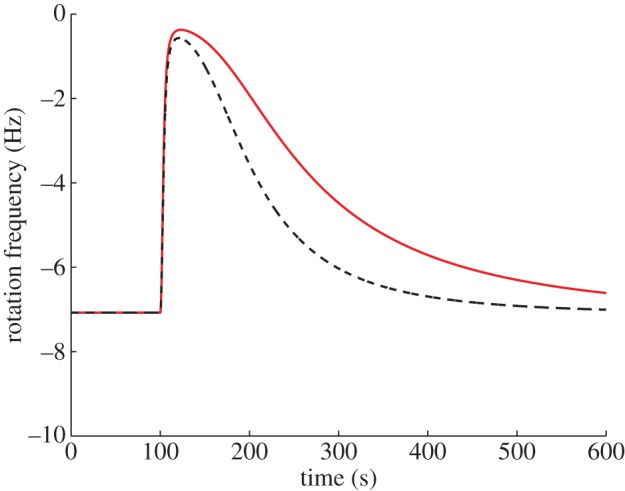
Simulations of the model in [9], subject to step changes in *L* from *L* = 1000 to 200 µM (red) and from *L* = 500 to 100 µM (black). (Online version in colour.)

In the preceding sections, we have shown that *R. sphaeroides* chemotaxis models based on the assumptions of §1.2 are able to reproduce experimental data as well as displaying FCD. By comparison, the model suggested in [9], based on different receptor dynamics, does not exhibit FCD in response to the inputs used in the simulation in figures 6 and 9 as shown in figure 12. Here, we propose the use of FCD as a transient dynamic phenotype to discriminate between the class of models we have presented and other models such as that in [9], and we outline a set of experiments with which to do this.

For our models to be valid, it is necessary that scalings of the ligand concentration signal sensed by the membrane cluster, *L*(*t*), yield the same chemotactic response whenever the conditions *K*_I_ ≪ *L* ≪ *K*_A_ and *K̃*_I_ ≪ *L̃* ≪ *K̃*_A_ are simultaneously satisfied. To the authors' best knowledge, the values of the four dissociation constants *K*_I_, *K*_A_, *K̃*_I_, *K̃*_A_ have not yet been experimentally determined (the values of these constants that were used for model fitting in the previous sections were adopted from the *E. coli* literature [4]). Furthermore, it is not precisely known how the signal *L* maps onto the signal *L̃*. For this reason, it is not yet possible to determine whether satisfaction of the condition *K*_I_ ≪ *L* ≪ *K*_A_ will necessarily mean that the corresponding condition on *L̃* will be met in order for FCD to be observed. Therefore, if FCD is not observed experimentally, this may be for one of at least three possible reasons we can suggest: the model and the MWC assumptions made in the preceding sections are incorrect, or the ligand concentration range in which FCD would be observed was not tested, or the individual clusters exhibit FCD in their kinase activities when their sensed ligands are within the above ranges but the pathway is such that when one of the conditions is satisfied, the other is not, resulting in no observed FCD in the flagellar chemotaxis response integrating signals from the two clusters.

To determine whether the last of these scenarios can be eliminated, we can test for the FCD range of the individual receptor clusters of the bacterium. From the ODEs describing the phosphotransfer network in the electronic supplementary material, it can be seen that deletion of the gene *cheA*_2_ would ensure that the only source of phosphorylation of the protein CheY_6_ would come from the cytoplasmic cluster. Simulations in the electronic supplementary material also show that even with this gene deletion, models I and II continue to display FCD in their levels of phosphorylated CheY_6_. By using FRET measurements as in [5], it would then be possible to search for the range of input ligand concentrations *L* that would lead to *L̃* being in the FCD range of the cytoplasmic cluster (if such a range exists). Similarly, deletion of the gene *cheA*_3_ would ensure that the only source of phosphorylation of the proteins CheY_3_, CheY_4_ and CheY_6_ is the membrane cluster, which displays FCD in these three proteins even with this mutation (see the electronic supplementary material). As before, the range of ligand concentrations *L* in which the polar cluster exhibits FCD could then be determined by FRET measurements. If these two tests yield ligand concentration ranges in which FCD is observed, then we would then expect the wild-type cell to show FCD behaviour at concentrations of 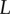 where these two ranges overlap. If such ranges exist for both clusters but do not overlap, or if such a range exists for only one of the clusters, we would then conclude that while FCD is not observed in the wild-type cell, such results lend support to the MWC model for the cluster(s) displaying the dynamic phenotype. Finally, if neither cluster displays an FCD concentration range, the models we have presented would be invalidated.

Based on observations of constant adaptation times, the work in [10] suggests that concentrations of the attractant ligand propionate in which FCD may be observed in *R. sphaeroides* lie between 4 µM and 12.5 mM, a wider range than that predicted by the *E. coli* dissociation constants we adopt for our models, *K*_I_ = 18 µM, *K*_A_ = 3 mM. The actual concentrations that yield FCD in *R. sphaeroides* are likely to lie somewhere between these two ranges. As shown in [5], even the more conservative of these estimates was sufficient for experimentally demonstrating FCD in *E. coli*.

An important feature to note about *R. sphaedoies* chemotaxis models satisfying the assumptions of §1.2 is that as long as the conditions of theorem A.2 are satisfied, the FCD property is preserved regardless of the exact dynamics in (1.2). It is also a property that is robust to variations in the structure of the interactions between the receptors and the chemotaxis proteins, and to variations in key parameters such as the receptor methylation and demethylation rates. If the experiments outlined earlier are able to show that the bacterium exhibits FCD behaviour, then the robustness predicted by our models can form the basis of a series of experiments to further validate the receptor dynamics proposed in §1.2. and to provide further ways of discriminating between models I, II, III on the one hand, and the model suggested in [9] on the other hand:

— if models I–III are to invalidate that of [9], then the wild-type bacterium, initially at steady state, should show near identical flagellar output response shapes to the step ligand inputs *L* = 1000 to 200 µM and *L* = 500 to 100 µM;
— overexpressing the chemotaxis protein CheY_4_ fivefold was shown in [8] to not destroy the chemotactic response of the bacterium. Such a mutant strain should therefore, according to models I–III, also exhibit FCD in response to a range of step changes in the external ligand concentration *L*. We can calculate this range for each of models I–III. In model I, the fivefold increase in CheY_4_ means that FCD should be observed within the range 7.92*K̃*_I_ ≪ *L* ≪ *K̃*_A_, whereas for models II, III this range is *K̃*_I_ ≪ *L* ≪ *K̃*_A_; and
— in [9], it was shown that deleting CheB_2_ reduced the average flagellar rotation frequency to zero, but the bacterium was still able to demonstrate a degree of response to ligand inputs. This mutant strain should therefore also demonstrate FCD within the range 2.64 *K̃*_I_ ≪ *L* ≪ *K̃*_A_ (for model I), and in the range (*K̃*_I_ ≪ *L* ≪ *K̃*_A_) (for models II and III).

Further model discrimination between models I–III can be performed using the tools presented in references [8,9]. For example, [8] suggest experiments with which to discriminate between two models, one of which features an interaction between the cytoplasmic cluster and CheY_3_, CheY_4_ and one which does not. In [9], experiments are suggested to discriminate between models on the basis of the connectivity of their CheB proteins with the two receptor clusters.

## 3. Discussion

The models presented herein differ from earlier *R. sphaeroides* chemotaxis models in two main respects: first, the receptor dynamics are based on the MWC allosteric model. This model has been shown to be a fairly accurate representation of the receptor dynamics in *E. coli*. The homologies between the bacteria and the similarities between their overall chemotaxis mechanisms give us reason to believe that the MWC model may, under experimental testing, eventually proved to be a realistic way of representing the *R. sphaeroides* receptor dynamics.

The second point of departure of these models from earlier ones is that the assumptions on the possible relationships between the external and internal ligand concentrations are relaxed to admit dynamic relations. The motivation behind this model is to capture any phase delays between sensed changes in the external ligand concentration and the effect of such changes on the internal cell environment.

The external–internal ligand relation of model I closely follows that of [9]. In effect, the activity of the cytoplasmic cluster depends on the external ligand concentration, *L*, and, indirectly, on the activity *a* of the membrane cluster via the phosphorylated chemotaxis proteins CheY_3_-P and CheY_4_-P, as schematically illustrated in figure 4. On the other hand, the cytoplasmic receptor activity in model II does not depend on any chemotaxis proteins, and its sensed ligand signals are merely phase-delayed versions of the external ligand concentration. As in model I, the external ligand signal in model III is conveyed to the cytoplasmic cluster via a static map.

**Figure 13.**
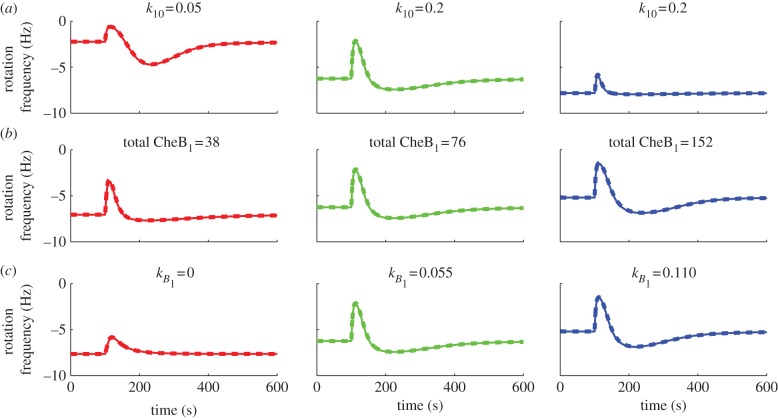
Simulations of model I subject to ligand step changes from *L* = 500 to 100 µM (solid lines) and *L* = 1000 to 200 µM (dashed lines) under parametric variations. (*a*) Variations in CheY_6_ dephosphorylation rate *k*_10_. (*b*) Variations in total CheB_1_ concentration. (*c*) Variations in demethylation rate *k*_*B*_1__. (Online version in colour.)

**Figure 14.**
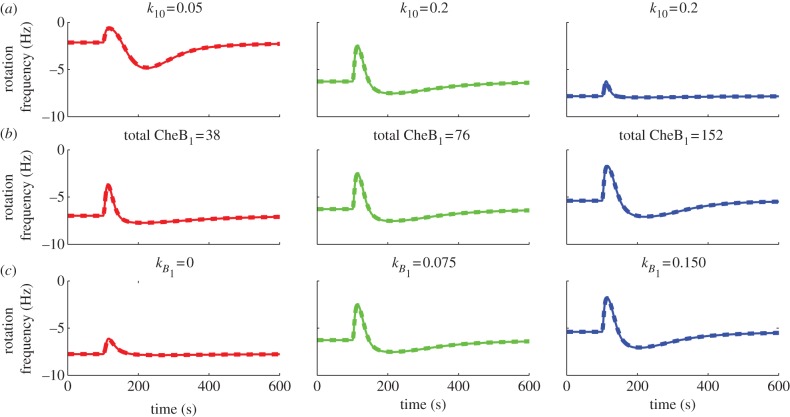
Simulations of model II subject to ligand step changes from *L* = 500 to 100 µM (solid lines) and *L* = 1000 to 200 µM (dashed lines) under parametric variations. (*a*) Variations in CheY_6_ dephosphorylation rate 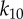. (*b*) Variations in total CheB_1_ concentration. (*c*) Variations in demethylation rate *k*_*B*_1__. (Online version in colour.)

The preceding section has shown that FCD is exhibited by all three models despite their structural differences. To illustrate the robustness of FCD to parametric variations, we simulated models I and II at different values of three different model parameters: the CheY_6_ auto-dephosphorylation rate *k*_10_, the total (phosphorylated and un-phosphorylated) CheB_1_ concentration and the demethylation rate *k*_*B*_1__ of the membrane cluster by CheB_1_. Figures 13 and 14 show simulations of models I and II under these parameter changes, each with steady-state initial conditions and subject to two step falls in ligand concentration that have the same fold change (from 500 to 100 µM, and from 1000 to 200 µM). As expected from theorem A.2, the parametric variations have no effect on FCD. The fact that these models can display FCD despite significant structural differences and parametric variations is in line with the evidence presented in Kojadinovic *et al.* [10] that bacterial strains having different cell architecture, protein expression levels and stoichiometries (arising from their being grown in different conditions) can each show constant adaptation times.

**Figure 15.**
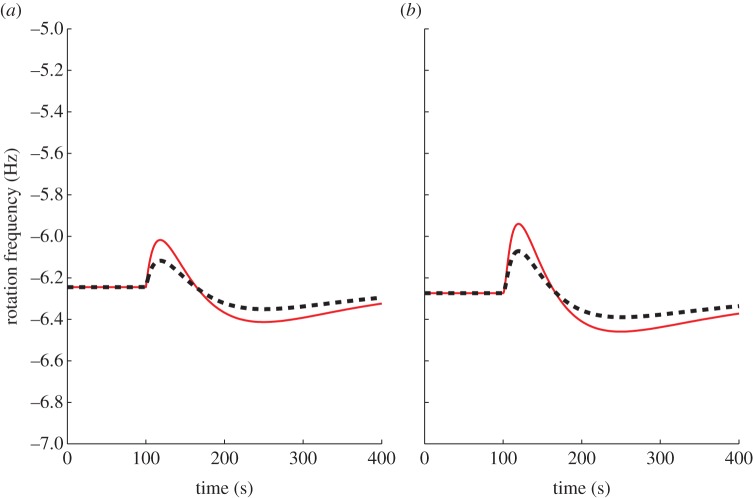
Outputs of models I (*a*) and II (*b*) in response to step changes in *L* from *L* = 1000 to 200 µM and from *L* = 500 to 100 µM with *K*_I_ = *K̃*_I_ = 1800 µM, *K*_A_ = *K̃*_A_ = 3000 µM. (Online version in colour.)

Parameters that would have an effect on the ligand ranges in which the models show FCD are the dissociation constants *K*_A_, *K*_I_, *K̃*_A_, *K̃*_I_, because approximation (1.6), which is assumed in theorem A.2, only holds in the ligand range *K*_I_ ≪ *L* ≪ *K*_A_ and *K̃*_I_ ≪ *L̃* ≪ *K̃*_A_. The fact that FCD is not seen outside this range is illustrated in figure 15 where *K*_I_ = *K̃*_I_ = 1800 µM, *K*_A_ = *K̃*_A_ = 3000 µM and where the models are subject to the same inputs as those in figures 6 and 9, which lie outside the FCD range of ligand concentrations.

As experimentally shown [6], chemotaxis requires CheY_6_ and one of either CheY_3_ or CheY_4_, as deletion of either CheY_6_ or both of CheY_3_ and CheY_4_ destroys the chemotactic ability of the bacterium. In the models we present, this was captured by the interaction of the three CheY proteins at the flagellum in what is effectively a AND logic gate that will only be activated if both CheY_6_ and at least one of CheY_3_*p*__ or CheY_4_*p*__ are present. The signal transduction dynamics [8,14] show that CheY_3_ and CheY_4_ are solely phosphorylated by the membrane cluster, whereas CheY_6_ receives most of its phosphates from the cytoplasmic cluster. In essence, this structure means that there are essentially two paths from the external ligands to the flagellum that terminate at the AND gate: one path via the membrane cluster in which CheY_3_*p*__ and CheY_4_*p*__ proteins convey the signal, and one path via the cytoplasmic cluster, in which CheY_6_*p*__ conveys the signal. This resembles a recurring biochemical motif [19], and the selective advantage it bestows could be improved *energy taxis* [6] with respect to simpler chemotaxis circuits such as that of *E. coli*. The main feature of this improved pathway is that the flagellar motion will vary only if both signalling paths from *L* to the flagellum are activated. Because the cytoplasmic cluster may integrate un-modelled metabolic information from within the cell, it would be important that any variation in flagellar activity only results from a change in the metabolic state of the cell that arises from a change in the local chemoeffector environment. If this is indeed the case, then the signalling path from the cytoplasmic cluster is activated only if the metabolic state of the cell changes, whereas the signalling path from the membrane cluster is activated only if the immediate chemical environment changes. Only if both are activated together would the cell ‘know’ that the change in its metabolic state is due to a change in chemoeffector concentration, and only then would it change its flagellar activity.

### 3.1. Chemotaxis in *R. sphaeroides* and in other bacteria

The chemotaxis pathway of *E. coli* has a well-characterized adaptation system composed of methyl-accepting chemotaxis protein receptors whose methylation levels are controlled through negative feedback. In *R. sphaeroides*, it is the transmembrane and cytoplasmic receptor clusters that are responsible for adaptation. Other bacteria, such as *Bacillus subtilis* also have multiple adaptation modules [20]. Despite this modularity, the work in this study, as well as that in references [8,9,15], models the adaptation mechanism in *R. sphaeroides* as having much more in common with that in *E. coli* than with *B. subtilis*. Indeed, the *R. sphaeroides* adaptation mechanism can, to some extent, be regarded as two coupled adaptation modules, the dynamics of each being such as those of *E. coli*. Because of this, each of the two receptor clusters is capable of demonstrating exact adaptation in its *kinase activity* independently of the other.^1^ By contrast, as discussed [20], none of the three adaptation systems in *B. subtilis* is able to do so on its own, and experimental observations suggests that at least two of the three are required for chemotaxis. There are further points of similarity between this paper's models of *R. sphaeroides* and the *E. coli* circuit that contrasts with the *B. subtilis* chemotaxis system: ligands act to inhibit the receptors' kinase activity, the CheY proteins cause the bacterium to stop swimming, and an increase in sensed ligand causes an increase in the average receptor methylation level. However, there is much that is unknown about the *R. sphaeroides* circuit, including, for example, what communication (if any) exists between the two clusters. The general model (1.3) allows for interactions between the chemotaxis proteins (represented by the vector **x**) and the receptors in a way that modifies the average kinase activities of the two clusters. An example of such an interaction exists in *B. subtilis* models, where the protein CheD promotes kinase activity, whereas CheV inhibits it, and, following [8,9], we also propose such an interaction in model I, whereby CheY_3_ and CheY_4_ promote the cytoplasmic cluster activity. Note that the presence of such an interaction will not affect the circuit's ability to display FCD, as long as the conditions of theorem A.2 are satisfied.

### 3.2. Selective advantage of fold-change detection

Whether FCD bestows upon the bacterium a selective advantage or simply arises as a by-product of the chemotaxis system's structure is a question of interest which has been addressed in [2]. The fact that FCD is present in simpler chemotaxis circuits than that of *R. sphaeroides* (e.g. in *E. coli*) suggests that the advantages gained by having such a property would be independent of the complexity of the bacterium's chemotaxis pathway. It may be that the metabolic payoff to the bacterium of moving to more chemically favourable regions depends on the relative chemical improvement in its environment rather than the absolute change. A reason for this could be that biasing its movement towards longer swims could be metabolically costly for the bacterium, and moving in this way is only worthwhile if the metabolic gain is significant. A potential disadvantage of FCD to the bacterium could be a high sensitivity to small fluctuations in sensed ligand when the background ligand concentration is low, due to the fact that the gain in the flagellar rotation frequency would then be high. However, this disadvantage is offset by the fact that FCD behaviour occurs only at background ligand concentrations significantly above a threshold, given by *K*_I_ in the models above.

### 3.3. Fold-change detection as a transient dynamic phenotype for model invalidation

The models we have presented provide an example of how transient dynamic phenotypes can be used to discriminate between competing biochemical models. Given two models of the same system, a mathematical analysis can be used to identify regions in the parameter and input spaces in which a certain qualitative dynamic behaviour, such as FCD, could be expected. Ideally, this behaviour would be expected to be robust to any genetic mutations or environmental conditions, and the conditions under which this behaviour would occur would be experimentally implementable. Model discrimination can then be performed on the basis of whether or not the system robustly reproduces the transient dynamic phenotype experimentally. This differs from traditional forms of model discrimination in that it can be used to discriminate between different biological mechanisms, and can be used to identify whether an observed phenomenon is due to the fine tuning of biological parameters or due to a more fundamental structural property of the system.

## Endnote

1 At the level of the bacterium's running and stopping motion, however, *R. sphaeroides* will cease to chemotax at all if the kinase activity of the transmembrane cluster is eliminated, for example by deleting *cheA*_2_. However, this occurs not because the cytoplasmic cluster depends on the transmembrane cluster for adaptation, but because this deletion would prevent the formation of any phosphorylated CheY_3_ and CheY_4_, which are necessary for chemotaxis.

## Appendix A

Assumption A.1. *The internalized ligand concentration* *L̃* *is related to the external ligand concentration* *L* *through a linear, time invariant filter*

*where*     *and* .
Theorem A.2. *Under assumption* A.1 *and approximation* (1.6), *if the chemotaxis system* (1.3) *has a unique steady state for any given* *L* *and steady-state initial conditions, it will exhibit adaptation and FCD* (*as defined in §2*) *in its activities* *a*, *ã* *for ligand inputs in the range* *K*_I_ ≪ *L* ≪ *K*_A_ *and* *K̃*_I_ ≪ *L̃* ≪ *K̃*_A_. Proof. In the following, we assume that all ligand concentrations lie in the ranges *K*_I_ ≪ *L* ≪ *K*_A_ and *K̃*_I_ ≪ *L̃* ≪ *K̃*_A_, and therefore approximation (1.6) holds. The proof is based on the existence of equivariances [3]. Suppose that in response to an external ligand input signal *L* = *L*_1_(*t*), the system (1.3), initially at a steady state corresponding to *L* = *L*_1_(0), exhibits a solution

and outputs *a*_1_(*t*) = *G*(*m*_1_(*t*),*L*_1_(*t*)), , . Now if the ligand input is scaled to *L* = *L*_2_(*t*) = *p**L*_1_(*t*), where , and if the initial state corresponds to *L* = *L*_2_(0), then

A1

is a solution of (1.3) because, under approximation (1.6), this implies that the outputs are

A2

which means that

A3

This, therefore, verifies the claim that if **m**_1_(*t*) is a solution of (1.3) when the ligand input is *L*(*t*) = *L*_1_(*t*), then **m**_2_(*t*) in (A 1) is a solution of (1.3) when the ligand input is *L*(*t*) = *p**L*_1_(*t*). Now, if **m**_1_(0) is a fixed point associated with *L* = *L*_1_(0), it holds that **m**_2_(0) is a fixed point when *L* = *L*_2_(0) = *p**L*_1_(0) since, from (A 2), *a*_2_(0) = *a*_1_(0) and *ã*_2_(0) = *ã*_1_(0), meaning that if  when *L* = *L*_1_(0) then, from (A 3),  when *L* = *L*_2_(0) = *p**L*_1_(0). Therefore, to prove that system (1.3) displays adaptation, we invoke the assumption that for each constant input *L* system (1.3) has a unique steady state. Then, if **m**_1_(0) is a steady state associated with *L* = *L*_1_(0), then it must be the unique steady state associated with that input. However, if **m**_1_(0) is a steady for input *L*_1_(0), then we know that **m**_2_(0) is the unique steady state associated with *L*_2_(0). Because the outputs associated with the two constant inputs *L*_1_(0), *L*_2_(0) are such that *a*_2_(0) = *a*_1_(0) and *ã*_2_(0) = *ã*_1_(0), it then follows that at constant inputs *L*, system (1.3) will always display the same output kinase activity. This proves adaptation. A similar argument can be used to prove FCD for (1.3). We have shown that if **m**_1_(*t*) is a solution of (1.3) when the ligand input is *L*(*t*) = *L*_1_(*t*), then **m**_2_(*t*) is a solution of (1.3) when the ligand input is *L*(*t*) = *p**L*_1_(*t*). Because the scaled inputs *L* = *L*_1_(*t*) and *L* = *L*_2_(*t*) = *p**L*_1_(*t*) yield the respective output pairs *a*_1_, *ã*_1_ and *a*_2_, *ã*_2_ and because, from (3.2), *a*_1_ = *a*_2_ and *ã*_1_ = *ã*_2_, it follows that system (1.3), under assumption A.1 exhibits FCD if the initial conditions of the system are **m**_1_(0) when *L* = *L*_1_ and **m**_2_(0) when *L* = *L*_2_(*t*) = *p**L*_1_(*t*). From the assumption that (1.3) has a unique fixed point for any constant *L*, it follows that if **m**_1_ (0) is the unique fixed point when *L* = *L*_1_(0), then **m**_2_(0) is the unique fixed point when *L* = *L*_2_(0) = *pL*_1_(0) because, from (A 2), this would mean that *a*_2_(0) = *a*_1_(0) and , implying that if  when *L* = *L*_1_(0) then, from (A 3),  when *L* = *L*_2_(0) = *pL*_1_(0). Therefore, if system (1.3) has a unique fixed point for any given *L*, starts from steady-state conditions and yields solution **m**_1_(t), then scaling *L* by *p* > 0 and initiating the system from steady-state conditions will cause the system to yield the solution **m**_2_(t) and thereby exhibit FCD. In the language of [3], we have proved that the mapping  is an equivariance associated to scalar symmetries on inputs.▪
